# Supplementary material for: Mechanistic investigations on Pinnick oxidation: a density functional theory study
Source: R Soc Open Sci. 2020 Feb 5;7(2):191568. doi: 10.1098/rsos.191568 (PMC7062072; doi:10.1098/rsos.191568)
Supplement: Supplementary information [file rsos191568supp1.docx]

Mechanistic Investigations on Pinnick Oxidation: A DFT Study

Aqeel A. Hussein,*^a,b^ Azzam A. M. Al-Hadedi,^c^ Alaa J. Mahrath,^d^ Gamal A. I. Moustafa,^b,e^ Faisal A. Almalki,^e^ Alaa Alqahtani,^e^ and Sergey Shityakov,^f^ Moaed E. Algazally^a^

^a.^ Faculty of Dentistry, University of Al-Ameed, Karbala P.O No: 198, Iraq. E-mail: aqeel_alaa85@yahoo.com

^b.^ Department of Chemistry, University of Southampton, Southampton, Hampshire, SO17 1BJ, United Kingdom. E-mail: aahh1f19@soton.ac.uk

^c.^ Department of Chemistry, Faculty of Science, University of Mosul, Mosul, Iraq.

^d.^ Chemistry and Biochemistry Department, College of Medicine, University of Babylon, Babylon, Iraq.

^e.^ Department of Medicinal Chemistry, Faculty of Pharmacy, Minia University, Egypt.

^f.^ Department of Pharmaceutical Chemistry, Faculty of Pharmacy, Umm Al-Qura University, Makkah 21955, Saudi Arabia.

^g.^ Department of Anesthesia and Critical Care, University of Würzburg, 97080 Würzburg, Germany.


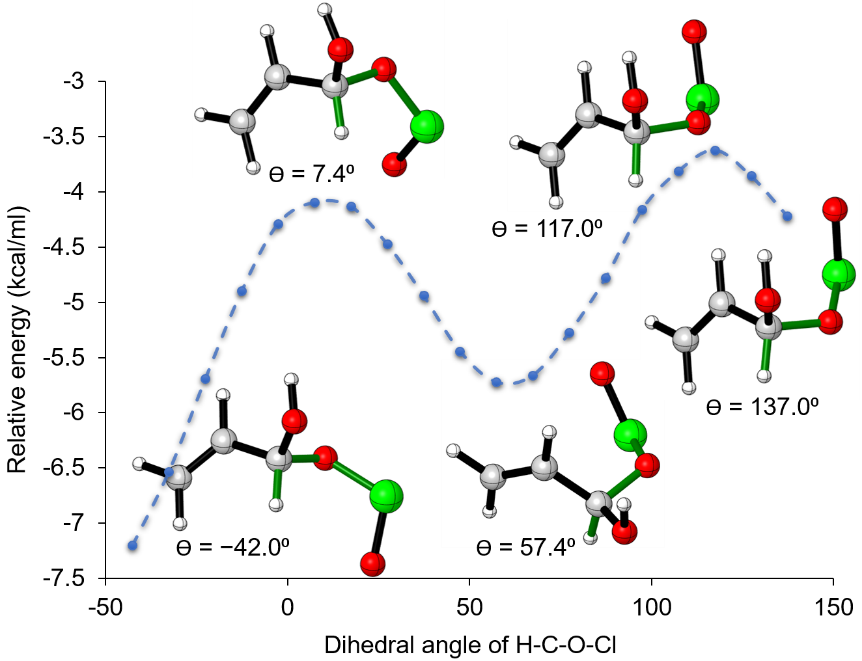


**Fig. SI1**: Scanning the dihedral angle, in degrees, of H-C-O-Cl highlighted in green.


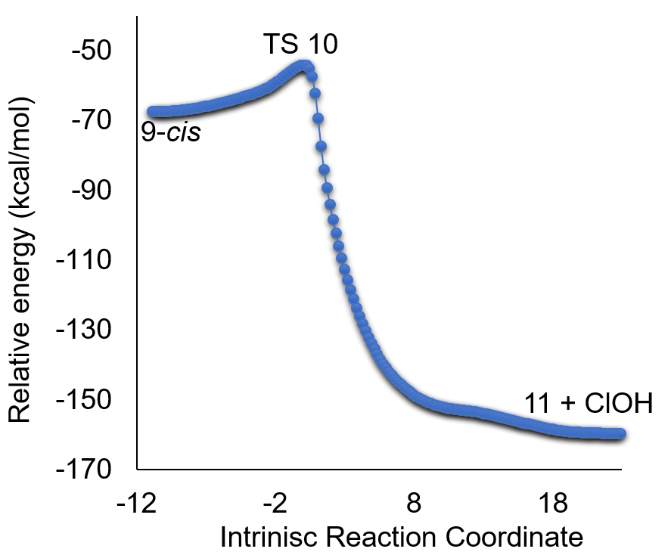


**Fig. SI2**: IRC results for TS **10**.


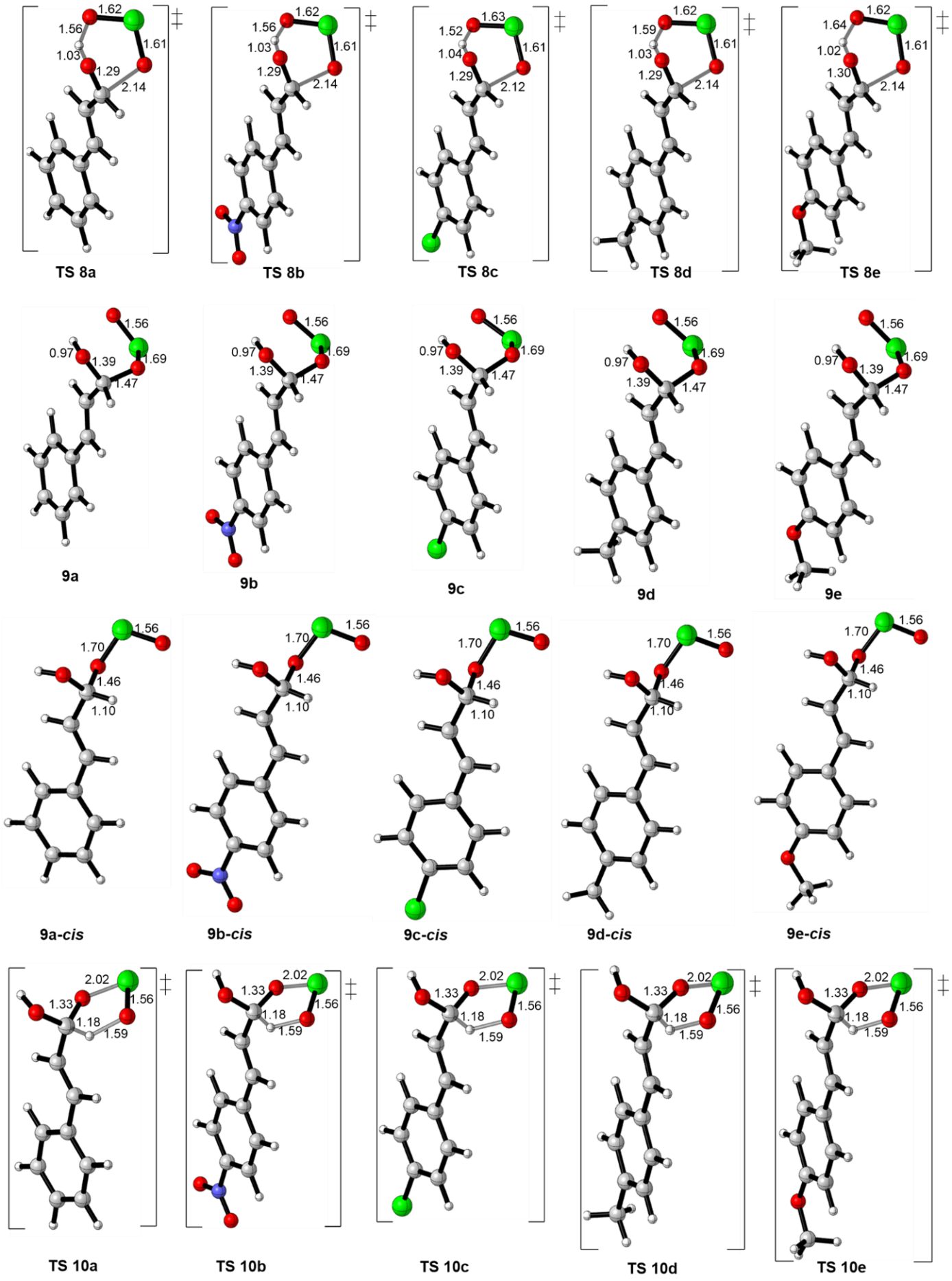


**Figure SI3**. Optimized geometries for the TSs and intermediates of the FRS and SRS involved in the oxidation of cinnamaldehyde and its derivatives.

**Supplementary data**

Cartesian coordinates, electronic energies and free energies of all stationary points during the reaction pathways. All provided geometries were optimized at the specified level of theory. All energies are in *t*-butanol at 298.15 K. All energies are in Hartree.

**7**

C 1.2153140 0.0667911 -0.0000065

H 2.1472200 -0.4982949 -0.0000105

H 1.2854560 1.1566571 -0.0000075

C 0.0231030 -0.5401179 0.0000025

H -0.0883860 -1.6257529 0.0000045

C -1.2030900 0.2701411 0.0000065

H -1.0528140 1.3701991 0.0000015

O -2.3268030 -0.1996229 0.0000095

E = -191.862025316, G = -191.826404

**7*_t_*_-BuOH_**

C -4.4574300 -0.7081680 -0.0976015

H -5.5196290 -0.7243250 0.1454105

H -3.9977120 -1.6461790 -0.4164855

C -3.7339080 0.4143420 -0.0146055

H -4.1562740 1.3702930 0.2987135

C -2.3095980 0.3792890 -0.3579065

H -1.9058030 -0.6058260 -0.6681925

O -1.5816760 1.3598330 -0.3202945

O 0.9398020 0.3848190 -1.2674555

C 1.7417170 0.0488860 -0.1247675

C 2.0658320 1.3203470 0.6559005

C 3.0083250 -0.5857300 -0.6796255

C 0.9833110 -0.9413830 0.7565295

H 2.5972290 2.0355990 0.0150135

H 2.6961850 1.0934660 1.5258175

H 1.1416000 1.7941490 1.0149365

H 0.0632380 -0.4853130 1.1471495

H 1.5985890 -1.2479940 1.6126095

H 0.7170400 -1.8374290 0.1810315

H 2.7633930 -1.4853190 -1.2594495

H 3.6846320 -0.8695720 0.1365535

H 3.5320860 0.1201150 -1.3372765

H 0.1290510 0.8161000 -0.9460045

E = -425.471781800, G = -425.311587

**7’*_t_*_-BuOH_**

C -0.3172644 -3.9049160 0.2193406

H -1.0750494 -4.2230560 0.9348576

H -0.0517554 -4.6035950 -0.5766294

C 0.2739076 -2.7073750 0.3021746

H 0.0374696 -1.9835100 1.0834496

C 1.2883096 -2.3404430 -0.6923704

H 1.5125426 -3.1035260 -1.4638324

O 1.8760046 -1.2695930 -0.7041414

O 0.8660126 0.7966370 1.0307706

C -0.0852904 1.5158900 0.2309996

C 0.6197806 2.1086280 -0.9863364

C -0.6327604 2.6157520 1.1289486

C -1.2000754 0.5684780 -0.2085934

H 1.4298276 2.7784400 -0.6702754

H -0.0866404 2.6808000 -1.6023384

H 1.0491786 1.3100040 -1.6066094

H -0.7954444 -0.2334690 -0.8411374

H -1.9607504 1.1080030 -0.7877814

H -1.6851504 0.1167560 0.6665826

H -1.1070284 2.1821420 2.0191136

H -1.3794524 3.2141600 0.5918406

H 0.1774946 3.2808790 1.4548206

H 1.2461346 0.0929140 0.4771476

E = -425.472254870, G = -425.310705

**TS 8**

C 1.9620434 -0.6073806 0.2922362

H 2.5839054 -0.8732906 1.1462492

H 2.3309274 -0.8592176 -0.7034158

C 0.7883814 0.0119364 0.4558682

H 0.3896554 0.2814414 1.4338332

C 0.0368304 0.4132144 -0.7271467

H 0.4455854 0.1455624 -1.7073228

O -0.8183226 1.3649014 -0.7199377

Cl -2.6798936 -0.5442266 -0.1244298

O -2.3412766 0.6001674 0.9908603

H -1.4253486 1.2916834 0.1784052

O -1.2724876 -1.2247916 -0.5151997

E = -802.872944588, G = -802.826485

Imaginary frequency = -445.35

**TS 8*_t_*_-BuOH_**

C 1.3046080 2.3823264 0.1430384

H 1.1383640 3.1233864 0.9237194

H 0.9983980 2.6368084 -0.8729026

C 1.8568020 1.1960894 0.4168074

H 2.1695350 0.9001634 1.4173764

C 1.9900760 0.2261394 -0.6625016

H 1.6947910 0.5321824 -1.6688296

O 2.0754710 -1.0369096 -0.4652066

Cl 4.7867790 -0.6777956 -0.3160486

O 3.9963020 -1.1424416 1.0332604

H 2.6909920 -1.2189336 0.3843324

O 4.0585410 0.6604564 -0.8427046

C -1.7730130 -0.4207286 -0.1389626

C -1.0389970 -1.1251446 0.9986884

H -0.2072390 -0.5083516 1.3622294

H -1.7234770 -1.3130016 1.8357454

H -0.6405220 -2.0912166 0.6593214

C -2.9282980 -1.2770006 -0.6479676

H -3.6548590 -1.4662566 0.1527324

H -3.4429100 -0.7712666 -1.4749806

H -2.5567880 -2.2462016 -1.0089676

C -2.2622160 0.9526104 0.2966314

H -1.4231720 1.5636944 0.6535074

H -2.7420160 1.4709434 -0.5433776

H -2.9937320 0.8555244 1.1083604

O -0.8558860 -0.1749996 -1.2223816

H -0.5175350 -1.0300776 -1.5209186

E = -1036.48222233, G = -1036.309744

Imaginary frequency = -400.17

**9**

C 1.6616996 -1.0693279 0.0959827

H 2.0735916 -1.7947189 0.7968517

H 2.1702006 -0.9455689 -0.8615883

C 0.5787356 -0.3576319 0.3996947

H 0.0798146 -0.4656649 1.3667937

C 0.0128686 0.6623641 -0.5424813

H 0.7054166 0.8870891 -1.3616653

O -0.3164494 1.8613661 0.0708567

Cl -2.2051174 -0.7560069 -0.3455393

O -2.7696364 0.1365681 0.8052887

H -0.8449514 1.6834181 0.8641847

O -1.1461724 0.1581141 -1.2883783

E = -802.900232348, G = -802.849701

**9-*cis***

C -1.8200915 -0.9727888 0.0657373

H -2.7899995 -1.1901848 -0.3809917

H -1.5145945 -1.5692648 0.9266423

C -1.0425805 -0.0084798 -0.4211907

H -1.3412995 0.5944612 -1.2827947

C 0.2793135 0.3377342 0.1935813

H 0.5091715 -0.2884368 1.0628313

O 0.3926955 1.6616302 0.5888753

Cl 2.8379975 0.2066252 -0.3194377

O 3.2080585 -1.0440978 0.5346093

H 0.0392115 2.2354872 -0.1079847

O 1.2421175 0.0373152 -0.8598777

E = -802.904400311, G = -802.855043

**TS 10**

C -1.4799811 -1.2477892 -0.0402758

H -2.4543741 -1.6222222 -0.3538578

H -0.8597071 -1.9014092 0.5753072

C -1.0699761 -0.0366242 -0.4035168

H -1.6866901 0.6251338 -1.0160618

C 0.2763139 0.5413888 0.0009532

H 0.7682209 -0.2572932 0.7098302

O 0.1797439 1.6987888 0.7902832

Cl 2.8444769 -0.1214582 -0.3554078

O 2.2555879 -0.7985112 0.9222462

H 0.1256709 2.4666438 0.2022332

O 1.1007139 0.6533518 -1.0317328

E = -802.882946887, G = -802.835405

Imaginary frequency = -569.94

**11**

C -0.8026726 1.3615372 0.1551458

H -0.7762706 2.4349702 -0.0291042

H -1.6890816 0.9434232 0.6337308

C 0.2203224 0.5810432 -0.1946252

H 1.1171014 0.9719902 -0.6736882

C 0.1743694 -0.8788118 0.0602558

O 1.2902514 -1.4937968 -0.3628712

H 1.2092714 -2.4439108 -0.1734202

O -0.7432916 -1.4764448 0.5845768

E = -267.111913300, G = -267.072692

**ClOH**

Cl 0.8490613 -0.9223107 -0.0000277

O -0.5779757 -0.0005237 -0.0000437

H -0.2710857 0.9228343 0.0000713

E = -535.935053833, G = -535.944716

**HOClO-*cis***

Cl 0.5007618 -0.7286968 -0.1543803

O -0.9636663 0.0889393 -0.2891233

H -1.2094243 0.3501152 0.6191337

O 1.6723288 0.2896422 -0.1756302

E = -611.023162084, G = -611.032198

**HOClO-*trans***

Cl 0.4598648 0.7066162 0.0000000

O -0.7002432 -0.5510797 0.0000000

H -1.5735612 -0.1082438 0.0000000

O 1.8139398 -0.0472927 0.0000000

E = -611.015307030, G = -611.025334

**7a**

C -1.0448175 0.8992694 -0.0001859

H -0.9384215 1.9887114 -0.0006759

C -2.2931085 0.3914384 0.0002841

H -2.5086795 -0.6775996 0.0007881

C -3.4337735 1.3035794 0.0001241

H -3.1886285 2.3859324 -0.0004309

O -4.5992045 0.9374684 0.0005281

C 0.2111685 0.1420084 -0.0001169

C 1.4240875 0.8470284 -0.0003589

C 0.2411795 -1.2630486 0.0001981

C 2.6415025 0.1677124 -0.0003039

H 1.4077785 1.9374714 -0.0005939

C 1.4558665 -1.9394866 0.0002591

H -0.6881135 -1.8312136 0.0004041

C 2.6592275 -1.2268126 0.0000081

H 3.5764115 0.7274704 -0.0004959

H 1.4684285 -3.0289086 0.0005081

H 3.6090965 -1.7610216 0.0000611

E = -422.870641944, G = -422.759293

**7b**

C -1.5278731 1.0860981 -0.0458890

H -1.4453461 2.1707351 -0.1591211

C -2.7591851 0.5547131 0.0591800

H -2.9539781 -0.5119809 0.1750920

C -3.9201031 1.4483871 0.0171260

H -3.6958471 2.5285511 -0.1015321

O -5.0740491 1.0647601 0.1038599

C -0.2533671 0.3559241 -0.0253480

C 0.9354459 1.0909341 -0.1486781

C -0.1886941 -1.0412319 0.1125830

C 2.1724279 0.4564111 -0.1361220

H 0.8893669 2.1739631 -0.2558001

C 1.0362809 -1.6917429 0.1272139

H -1.0992241 -1.6288189 0.2094270

C 2.1944739 -0.9265399 0.0021550

H 3.0968989 1.0198831 -0.2312401

H 1.0986119 -2.7715429 0.2333790

N 3.4926979 -1.6133179 0.0172760

O 4.5017679 -0.9397809 -0.0923381

O 3.4996959 -2.8254049 0.1387770

E = -627.342921036, G = -627.231659

**7c**

C -1.0779166 0.9101402 -0.0002860

H -0.9673016 1.9987192 -0.0010600

C -2.3280356 0.4080332 0.0004580

H -2.5525066 -0.6591998 0.0012620

C -3.4597566 1.3327402 0.0002030

H -3.2016536 2.4122572 -0.0006500

O -4.6292286 0.9817362 0.0008400

C 0.1770034 0.1522382 -0.0001960

C 1.3880054 0.8591212 -0.0003080

C 0.2120334 -1.2521098 0.0000330

C 2.6109624 0.1931282 -0.0002260

H 1.3742504 1.9489962 -0.0004660

C 1.4231064 -1.9325398 0.0001290

H -0.7128016 -1.8265228 0.0001430

C 2.6089704 -1.1977058 -0.0000050

H 3.5484934 0.7455372 -0.0003150

H 1.4499014 -3.0204068 0.0003120

Cl 4.1364734 -2.0541618 0.0001320

E = -882.466377707, G = -882.366528

**7d**

C -1.6765431 1.2246516 -0.0023966

H -1.5598761 2.3130576 -0.0048176

C -2.9322741 0.7322946 -0.0041176

H -3.1622121 -0.3337514 -0.0017856

C -4.0570331 1.6614376 -0.0086116

H -3.7929601 2.7395786 -0.0109016

O -5.2301841 1.3183546 -0.0097566

C -0.4283831 0.4596326 0.0024854

C 0.7903859 1.1537186 0.0060284

C -0.3985131 -0.9453864 0.0075924

C 2.0022299 0.4678426 0.0133724

H 0.7841539 2.2442626 0.0062434

C 0.8136749 -1.6236334 0.0146904

H -1.3280501 -1.5131684 0.0096244

C 2.0346179 -0.9309304 0.0145654

H 2.9379539 1.0275926 0.0199204

H 0.8179309 -2.7140364 0.0225654

C 3.3417389 -1.6751474 -0.0073376

H 3.6552959 -1.8682934 -1.0432026

H 4.1348479 -1.0940404 0.4776304

H 3.2531999 -2.6440354 0.4982094

E = -462.172760989, G = -462.036056

**7e**

C -1.9090594 1.2717139 -0.2109238

H -1.7471694 2.3375049 -0.0197978

C -3.1608734 0.8780699 -0.5302478

H -3.4298724 -0.1579421 -0.7393278

C -4.2221234 1.8728859 -0.6048278

H -3.9203894 2.9188009 -0.3874298

O -5.3850204 1.6168009 -0.8853238

C -0.7260384 0.4252969 -0.0889888

C 0.4996196 1.0093569 0.2530392

C -0.7635724 -0.9677321 -0.3024488

C 1.6630806 0.2518849 0.3841042

H 0.5476026 2.0856549 0.4219012

C 0.3794326 -1.7339271 -0.1769318

H -1.6994474 -1.4564581 -0.5699278

C 1.6014546 -1.1286891 0.1675272

H 2.5956926 0.7417089 0.6510372

H 0.3559116 -2.8103311 -0.3407038

O 2.6626596 -1.9628151 0.2660322

C 3.9205896 -1.3908411 0.6135652

H 4.6276476 -2.2236141 0.6387542

H 4.2375816 -0.6599811 -0.1423648

H 3.8722926 -0.9173481 1.6032842

E = -537.371153861, G = -537.228540

**TS 8a**

C 0.1505383 0.0522172 -0.8944906

H 0.5138373 -0.2556768 -1.8788646

C -1.0173087 0.7269672 -0.8348596

H -1.4691017 1.0814452 0.0904904

C -1.6883977 1.0347702 -2.0708186

H -1.2322127 0.7183592 -3.0122756

O -2.5685867 1.9714112 -2.1827076

Cl -4.4527707 0.0727592 -1.5446886

O -4.1825307 1.2299002 -0.4375146

H -3.1520917 1.9954112 -1.3298036

O -3.0302877 -0.6144648 -1.8691536

C 1.0100433 -0.3068568 0.2332914

C 2.2271053 -0.9521548 -0.0365956

C 0.6629373 -0.0299208 1.5673274

C 3.0853893 -1.3095748 1.0010814

H 2.4961183 -1.1703588 -1.0706156

C 1.5201043 -0.3890268 2.6008504

H -0.2795717 0.4635322 1.8003574

C 2.7325963 -1.0280588 2.3207004

H 4.0287863 -1.8076438 0.7804744

H 1.2450723 -0.1745438 3.6328214

H 3.4003313 -1.3084918 3.1349944

E = -1033.88212796, G = -1033.756529

Imaginary frequency = -357.64

**TS 8b**

C -0.1863456 0.2460039 -1.2209775

H 0.2040724 0.0348059 -2.2196795

C -1.3881626 0.8420939 -1.1175075

H -1.8625286 1.1028059 -0.1723545

C -2.0733016 1.2120769 -2.3412915

H -1.6029856 0.9581939 -3.2955865

O -2.9616446 2.1363229 -2.3920575

Cl -4.7934926 0.1761979 -1.8630925

O -4.5364836 1.3177349 -0.7267795

H -3.5870966 2.0674239 -1.5299615

O -3.3545076 -0.4673931 -2.2043765

C 0.6840364 -0.1451411 -0.1060165

C 1.9444434 -0.6824891 -0.4076065

C 0.2964224 -0.0040111 1.2377455

C 2.8158734 -1.0681241 0.6043915

H 2.2451404 -0.7981911 -1.4481925

C 1.1505564 -0.3893551 2.2603295

H -0.6786266 0.4061539 1.4925695

C 2.3965074 -0.9121811 1.9202935

H 3.7953784 -1.4828371 0.3811525

H 0.8605174 -0.2873131 3.3027655

N 3.3068314 -1.3161721 3.0005105

O 4.4122144 -1.7276691 2.6966535

O 2.9131814 -1.2189361 4.1490685

E = -1238.35378286, G = -1238.229185

Imaginary frequency = -398.55

**TS 8c**

C 0.1364591 0.0661461 -0.9137730

H 0.5105951 -0.2241349 -1.8993750

C -1.0382939 0.7260231 -0.8503370

H -1.4987349 1.0631301 0.0773330

C -1.7035789 1.0465081 -2.0895990

H -1.2393399 0.7346591 -3.0287570

O -2.5723779 1.9919291 -2.1990550

Cl -4.4691289 0.1062911 -1.5972740

O -4.1962829 1.2655341 -0.4884380

H -3.1777399 2.0005921 -1.3488310

O -3.0472359 -0.5888649 -1.9098930

C 0.9922761 -0.2996189 0.2148880

C 2.2132081 -0.9356419 -0.0538720

C 0.6399321 -0.0397219 1.5502450

C 3.0740421 -1.3043319 0.9758230

H 2.4942491 -1.1424329 -1.0864840

C 1.4867981 -0.4028799 2.5891010

H -0.3027129 0.4495291 1.7899560

C 2.6958911 -1.0300639 2.2860810

H 4.0214891 -1.7958379 0.7637380

H 1.2149071 -0.2046169 3.6239490

Cl 3.7655791 -1.4821959 3.5945730

E = -1493.47777501, G = -1493.365522

Imaginary frequency = -368.60

**TS 8d**

C -0.9965928 0.9992335 0.0113062

H -1.1081988 2.0306115 -0.3356758

C -2.1012888 0.3615935 0.4590642

H -2.1052318 -0.6637675 0.8260712

C -3.3366478 1.0944855 0.4928622

H -3.3431068 2.1348235 0.1596362

O -4.3391978 0.7609005 1.2370052

Cl -5.2330298 -0.7342275 -0.8888128

O -4.7129888 -1.5691105 0.4023882

H -4.4194778 -0.2630825 1.2599122

O -4.0322798 0.2451595 -1.3390898

C 0.3593442 0.4636515 -0.0478898

C 1.3899132 1.3020105 -0.4961198

C 0.6778032 -0.8555025 0.3270182

C 2.7031282 0.8421685 -0.5662918

H 1.1545062 2.3256535 -0.7895778

C 1.9869152 -1.3061245 0.2532122

H -0.1012588 -1.5323945 0.6754922

C 3.0225672 -0.4661845 -0.1927788

H 3.4905682 1.5096615 -0.9165358

H 2.2208472 -2.3312535 0.5430292

C 4.4334072 -0.9804075 -0.2587838

H 4.4911032 -1.8688795 -0.9016428

H 4.7824062 -1.2784645 0.7391882

H 5.1167892 -0.2205545 -0.6529858

E = -1073.18475975, G = -1073.035608

Imaginary frequency = -355.53

**TS 8e**

C -1.2797490 0.8694828 0.0121685

H -1.3452000 1.9215158 -0.2804915

C -2.4369750 0.2225968 0.2906115

H -2.4921210 -0.8231982 0.5896295

C -3.6563280 0.9705958 0.2201605

H -3.6135150 2.0306058 -0.0391135

O -4.7517750 0.6086178 0.8099865

Cl -5.4052810 -0.7007972 -1.5203715

O -5.0587880 -1.6625682 -0.2634075

H -4.8417670 -0.4036862 0.7664965

O -4.1390270 0.2745428 -1.7422895

C 0.0661880 0.3252058 0.0733345

C 1.1455300 1.1736918 -0.2094325

C 0.3391400 -1.0185232 0.4070805

C 2.4624370 0.7241668 -0.1643585

H 0.9480720 2.2141748 -0.4693985

C 1.6394020 -1.4803632 0.4559295

H -0.4749470 -1.7075462 0.6281755

C 2.7106050 -0.6117542 0.1725135

H 3.2727400 1.4117848 -0.3902235

H 1.8602700 -2.5161382 0.7095815

O 3.9434540 -1.1585142 0.2498815

C 5.0577890 -0.3131622 -0.0269985

H 5.9446560 -0.9391932 0.0965805

H 5.0113430 0.0634248 -1.0573995

H 5.0938480 0.5250368 0.6813545

E = -1148.38330393, G = -1148.233069

Imaginary frequency = -344.19

**TS 9a**

C 0.2540154 0.4752371 -0.7476666

H 0.7884084 0.6713801 -1.6814526

C -0.8745956 1.1546831 -0.5122396

H -1.4541926 1.0337281 0.4056954

C -1.3814386 2.1684251 -1.4888646

H -0.6257266 2.4296241 -2.2385426

O -1.8104866 3.3471041 -0.8970646

Cl -3.5591086 0.6646091 -1.5456836

O -4.2927436 1.5074211 -0.4535496

H -2.3806206 3.1414421 -0.1408266

O -2.4488076 1.6418351 -2.3538396

C 0.8771394 -0.5307809 0.1301944

C 2.0717864 -1.1328359 -0.2897946

C 0.3262964 -0.9130849 1.3644984

C 2.7045174 -2.0930849 0.4989424

H 2.5077424 -0.8430909 -1.2465896

C 0.9576104 -1.8711969 2.1511544

H -0.5996776 -0.4614889 1.7182874

C 2.1487774 -2.4651649 1.7222134

H 3.6317704 -2.5515159 0.1563084

H 0.5191724 -2.1588379 3.1063334

H 2.6401624 -3.2144069 2.3424864

E = -1033.90670169, G = -1033.780547

**9a-*cis***

C 0.3903843 -0.3484812 0.3409687

H 0.6980903 -1.2896392 0.8048877

C 1.3300313 0.5744268 0.1044157

H 1.1150483 1.5421568 -0.3539363

C 2.7574123 0.3283978 0.4772467

H 2.8920753 -0.6343142 0.9828337

O 3.3236533 1.3036608 1.2844947

Cl 5.0666823 -0.1992142 -0.6313353

O 5.1071723 -1.7301542 -0.3404223

H 3.0931143 2.1799188 0.9408837

O 3.4477143 0.2710148 -0.8079593

C -1.0490057 -0.2391402 0.0469947

C -1.8822057 -1.3100032 0.4004527

C -1.6195317 0.8854688 -0.5712653

C -3.2524827 -1.2641742 0.1460337

H -1.4463137 -2.1870882 0.8802477

C -2.9865797 0.9305998 -0.8259443

H -0.9961027 1.7318348 -0.8575603

C -3.8083107 -0.1432592 -0.4688643

H -3.8845547 -2.1060122 0.4273047

H -3.4181057 1.8076998 -1.3074723

H -4.8781857 -0.1036982 -0.6720043

E = -1033.91066037, G = -1033.783177

**9b**

C -0.0326262 0.7737900 -1.0345966

H 0.4765438 0.9466610 -1.9858096

C -1.1505322 1.4584550 -0.7698016

H -1.6975872 1.3486640 0.1694564

C -1.6866102 2.4686670 -1.7378186

H -0.9689552 2.6930640 -2.5347496

O -2.0391972 3.6692590 -1.1405966

Cl -3.8950602 1.0267270 -1.6127186

O -4.4434062 1.8833050 -0.4261306

H -2.6014562 3.4937800 -0.3701666

O -2.8195252 1.9596020 -2.5186136

C 0.6073928 -0.2167240 -0.1510996

C 1.7769338 -0.8463310 -0.6022786

C 0.0964728 -0.5494490 1.1151514

C 2.4322678 -1.7897290 0.1807814

H 2.1777618 -0.5911750 -1.5826396

C 0.7363988 -1.4878660 1.9113754

H -0.8075982 -0.0750910 1.4914234

C 1.8953348 -2.0920150 1.4265374

H 3.3386408 -2.2789380 -0.1664096

H 0.3484208 -1.7516340 2.8918734

N 2.5743548 -3.0868140 2.2633334

O 3.5833348 -3.6135390 1.8275884

O 2.0986968 -3.3426680 3.3559094

E = -1238.38002312, G = -1238.253329

**9b-*cis***

C -0.5819046 -0.6201777 -0.6138160

H -1.4987536 -0.5026997 -1.1964120

C 0.1114784 -1.7573307 -0.7320480

H 1.0316714 -1.9563807 -0.1786090

C -0.3490076 -2.8570957 -1.6377210

H -1.3166936 -2.6363867 -2.1017150

O -0.4512326 -4.0949127 -1.0245580

Cl 0.2705944 -3.9659597 -3.9264290

O -0.8135406 -3.3051277 -4.8290040

H 0.3379774 -4.2515337 -0.4837200

O 0.6698524 -2.8851727 -2.6810670

C -0.2349466 0.5197273 0.2526300

C -1.1328006 1.5953703 0.3260950

C 0.9566784 0.5716513 0.9960790

C -0.8657746 2.6990583 1.1282320

H -2.0553816 1.5646853 -0.2523370

C 1.2420284 1.6652113 1.8001230

H 1.6766214 -0.2428617 0.9469870

C 0.3205064 2.7097813 1.8527010

H -1.5605006 3.5327603 1.1904650

H 2.1626534 1.7137293 2.3759200

N 0.6161514 3.8656583 2.7054060

O -0.2011256 4.7683703 2.7573790

O 1.6654484 3.8696363 3.3254200

E = -1238.38434658, G = -1238.258236

**9c**

C 0.2628541 0.5254600 -0.7623375

H 0.8190501 0.7488250 -1.6766915

C -0.8820829 1.1791720 -0.5341745

H -1.4814239 1.0134760 0.3642975

C -1.3895189 2.2185770 -1.4837895

H -0.6195149 2.5414600 -2.1930795

O -1.8793549 3.3514270 -0.8508155

Cl -3.4971569 0.6398690 -1.6647825

O -4.2113319 1.3769700 -0.4859415

H -2.4925399 3.0849150 -0.1484375

O -2.4120399 1.6984350 -2.4034385

C 0.8712961 -0.4925050 0.1114605

C 2.0262881 -1.1521360 -0.3290975

C 0.3445341 -0.8257660 1.3695765

C 2.6457451 -2.1259510 0.4521745

H 2.4480881 -0.9036010 -1.3029305

C 0.9507921 -1.7917660 2.1639365

H -0.5473199 -0.3268800 1.7458875

C 2.0961771 -2.4315330 1.6919565

H 3.5413061 -2.6366010 0.1028245

H 0.5427011 -2.0432960 3.1410855

Cl 2.8634511 -3.6485500 2.6923165

E = -1493.5024507, G = -1493.388598

**9c-*cis***

C 0.5010709 0.1417942 -0.4082883

H 1.3974689 -0.1432718 -0.9655323

C -0.2101601 1.1895922 -0.8401283

H -1.1250091 1.5309142 -0.3507013

C 0.2100729 1.9739042 -2.0420073

H 1.1841569 1.6562612 -2.4298103

O 0.2659999 3.3439382 -1.8424933

Cl -0.4434071 2.3111382 -4.5547343

O 0.7203609 1.4912142 -5.1899303

H -0.5226071 3.6304752 -1.3571913

O -0.8065421 1.6448022 -3.0381503

C 0.1972159 -0.6972758 0.7635457

C 0.9852629 -1.8326228 0.9959627

C -0.8465591 -0.4095868 1.6573357

C 0.7423699 -2.6731578 2.0805667

H 1.8026519 -2.0675728 0.3141797

C -1.1011701 -1.2343178 2.7468977

H -1.4729811 0.4694432 1.5141597

C -0.3018051 -2.3599588 2.9429887

H 1.3568789 -3.5551778 2.2510797

H -1.9107381 -1.0071028 3.4379467

Cl -0.6225301 -3.4034328 4.3143037

E = -1493.50665296, G = -1493.390486

**9d**

C -0.0836790 0.9651722 -1.0814125

H 0.4653480 1.1696512 -2.0050395

C -1.2190290 1.6396592 -0.8601485

H -1.8127980 1.5125702 0.0481705

C -1.7216020 2.6456802 -1.8461265

H -0.9475110 2.9428432 -2.5626335

O -2.2202700 3.8014442 -1.2630625

Cl -3.8667710 1.1108292 -1.9797385

O -4.6366690 1.9637522 -0.9194325

H -2.8448420 3.5618702 -0.5614265

O -2.7327770 2.0864222 -2.7578635

C 0.5264540 -0.0457938 -0.2032925

C 1.7252710 -0.6487808 -0.6018675

C -0.0434050 -0.4407658 1.0194465

C 2.3388290 -1.6191038 0.1915165

H 2.1822200 -0.3555178 -1.5478865

C 0.5715260 -1.4064078 1.8049465

H -0.9748000 0.0083472 1.3626775

C 1.7726420 -2.0152328 1.4049735

H 3.2720680 -2.0751848 -0.1406035

H 0.1140000 -1.6993508 2.7510655

C 2.4139370 -3.0690878 2.2661335

H 1.7745240 -3.9602848 2.3286865

H 3.3866020 -3.3721848 1.8631005

H 2.5607320 -2.7005468 3.2898175

E = -1073.20849989, G = -1073.058706

**9d*-cis***

C -0.5243570 -0.7607629 -0.8141406

H -1.4595900 -0.6678689 -1.3733266

C 0.2813960 -1.7909609 -1.0998246

H 1.2321550 -1.9631939 -0.5905566

C -0.0871100 -2.7896489 -2.1495806

H -1.0724560 -2.5897989 -2.5848086

O -0.0812400 -4.1102769 -1.7255966

Cl 0.6041540 -3.5073569 -4.5726596

O -0.5734650 -2.8433729 -5.3490016

H 0.7159700 -4.2759079 -1.2003216

O 0.9224270 -2.5858279 -3.1855096

C -0.2936020 0.2862231 0.1938844

C -1.2794210 1.2615331 0.3855164

C 0.8748560 0.3586831 0.9719744

C -1.1093880 2.2796551 1.3241534

H -2.1924050 1.2203531 -0.2097416

C 1.0399250 1.3757241 1.9028194

H 1.6637050 -0.3830449 0.8503204

C 0.0524240 2.3560951 2.0952864

H -1.8929060 3.0265761 1.4552184

H 1.9545020 1.4176381 2.4961794

C 0.2599840 3.4571581 3.0994404

H 1.0856640 4.1129661 2.7907664

H -0.6421230 4.0698901 3.2063694

H 0.5209000 3.0455271 4.0831384

E = -1073.21267024, G = -1073.059386

**9e**

C -0.3605884 1.0770427 -1.1287169

H 0.2456506 1.2941247 -2.0129899

C -1.5688914 1.6487327 -1.0381819

H -2.2267284 1.5014247 -0.1784559

C -2.0731864 2.5551667 -2.1147159

H -1.2681324 2.9025547 -2.7723469

O -2.7413694 3.6750937 -1.6411469

Cl -4.0266374 0.7952597 -2.3675109

O -4.9708144 1.6018547 -1.4173979

H -3.4001084 3.4003197 -0.9851129

O -2.9303344 1.8545217 -3.0871239

C 0.2607396 0.1657847 -0.1584309

C 1.5273526 -0.3562543 -0.4378469

C -0.3638744 -0.2195133 1.0433531

C 2.1694186 -1.2393123 0.4322381

H 2.0282686 -0.0710753 -1.3636419

C 0.2579626 -1.0923093 1.9186431

H -1.3499394 0.1666417 1.2988811

C 1.5291236 -1.6104693 1.6172091

H 3.1524306 -1.6248233 0.1755471

H -0.2230944 -1.3911533 2.8491281

O 2.0532426 -2.4624053 2.5342341

C 3.3288126 -3.0273893 2.2489081

H 3.5600906 -3.6821483 3.0927931

H 3.2950916 -3.6167613 1.3225301

H 4.0955156 -2.2449073 2.1701541

E = -1148.40620162, G = -1148.250622

**9e-*cis***

C 0.4617748 1.0985224 -0.7747094

H 1.4271148 1.0127084 -1.2816814

C -0.2873332 2.1802754 -1.0268564

H -1.2671262 2.3454764 -0.5734874

C 0.1780278 3.2405504 -1.9707154

H 1.2193148 3.0974234 -2.2799074

O 0.0572328 4.5367564 -1.4949884

Cl -0.2139622 4.0627814 -4.4290264

O 1.0868268 3.4906414 -5.0725234

H -0.8059292 4.6410214 -1.0667264

O -0.6728422 3.0525594 -3.1468064

C 0.1292228 -0.0175276 0.1209066

C 1.0106208 -1.0999316 0.2037486

C -1.0417452 -0.0569156 0.9021756

C 0.7534768 -2.2006176 1.0230866

H 1.9241278 -1.0880486 -0.3918394

C -1.3112812 -1.1375596 1.7232356

H -1.7532822 0.7673914 0.8724876

C -0.4161092 -2.2189376 1.7869896

H 1.4632558 -3.0229646 1.0538246

H -2.2156332 -1.1698996 2.3295626

O -0.7710852 -3.2317116 2.6176446

C 0.1053488 -4.3511946 2.6941596

H -0.3574172 -5.0444386 3.4010746

H 0.2038478 -4.8367886 1.7137926

H 1.0935548 -4.0495726 3.0665796

E = -1148.41036546, G =-1148.252173

**TS 10a**

C -0.3356015 -0.2034640 -0.4382837

H -1.1676335 0.0277250 -1.1091857

C 0.3809985 -1.3081530 -0.6658437

H 1.2230435 -1.6124600 -0.0418897

C 0.0813105 -2.2445130 -1.8205007

H -0.8634375 -1.8005070 -2.3612317

O -0.3192275 -3.5322270 -1.4251387

Cl 0.0649535 -2.0210610 -4.4898047

O -1.3209505 -1.6418710 -3.8774497

H 0.4745025 -4.0728350 -1.2997447

O 1.0454435 -2.2341920 -2.7323807

C -0.1208655 0.7667520 0.6522503

C -1.0054345 1.8482760 0.7687033

C 0.9311605 0.6545580 1.5759313

C -0.8494045 2.7945470 1.7816873

H -1.8252455 1.9451140 0.0556353

C 1.0865425 1.5979920 2.5867403

H 1.6364025 -0.1727770 1.5068683

C 0.1973505 2.6719330 2.6942003

H -1.5463895 3.6287940 1.8573263

H 1.9082725 1.4990540 3.2957283

H 0.3242105 3.4093160 3.4863833

E = -1033.88867979, G = -1033.766041

Imaginary frequency = -561.50

**TS 10b**

C -0.4049446 -0.6906648 -0.6530730

H -1.2672296 -0.5866348 -1.3166470

C 0.3001674 -1.8246698 -0.6870700

H 1.1697964 -2.0115748 -0.0546420

C -0.0570506 -2.9667468 -1.6197870

H -1.0670586 -2.6589048 -2.1371080

O -0.3572086 -4.1658408 -0.9555300

Cl -0.3218086 -3.2260808 -4.2710290

O -1.6611626 -2.7783568 -3.6063670

H 0.4673064 -4.6558988 -0.8202730

O 0.8199174 -3.0938308 -2.6075020

C -0.1351886 0.4713152 0.2124790

C -0.9979726 1.5745662 0.1260580

C 0.9408614 0.5159012 1.1160190

C -0.8038936 2.7023672 0.9151750

H -1.8339236 1.5461752 -0.5719710

C 1.1509324 1.6319822 1.9122150

H 1.6257764 -0.3249878 1.2029050

C 0.2720004 2.7076172 1.7956120

H -1.4714876 3.5577542 0.8507420

H 1.9799034 1.6740442 2.6138880

N 0.4926044 3.8902662 2.6324350

O -0.2782196 4.8279522 2.5211370

O 1.4378834 3.8842512 3.4023330

E = -1238.36281691, G = -1238.23942

Imaginary frequency = -558.85

**TS 10c**

C 0.3333149 0.1674454 -0.4850491

H 1.1495849 -0.1499186 -1.1395361

C -0.3606311 1.2618004 -0.8104261

H -1.1855311 1.6477994 -0.2091431

C -0.0561241 2.0716214 -2.0560491

H 0.8799769 1.5620924 -2.5529431

O 0.3623379 3.3868734 -1.7948431

Cl -0.0579001 1.5750054 -4.6878041

O 1.3243089 1.2367334 -4.0449811

H -0.4228511 3.9510864 -1.7380811

O -1.0252621 1.9804284 -2.9577111

C 0.1132399 -0.6840546 0.6982739

C 0.9679689 -1.7750756 0.9056319

C -0.9136791 -0.4506256 1.6267209

C 0.8164059 -2.6157266 2.0069769

H 1.7695589 -1.9710486 0.1931109

C -1.0798721 -1.2779166 2.7315349

H -1.5986831 0.3854164 1.4948249

C -0.2089541 -2.3523156 2.9074999

H 1.4850089 -3.4606626 2.1608909

H -1.8770691 -1.0935326 3.4494449

Cl -0.4151481 -3.3954246 4.3016569
E = -1493.48476083, G = -1493.371396

Imaginary frequency = -565.75

**TS 10d**

C -0.3628670 -0.7889430 -0.8742345

H -1.2409300 -0.6818940 -1.5174415

C 0.4437520 -1.8376740 -1.0652075

H 1.3375890 -2.0200820 -0.4662435

C 0.1823710 -2.8752350 -2.1396945

H -0.8192940 -2.5533710 -2.6639475

O -0.0849800 -4.1627990 -1.6432895

Cl 0.0360820 -2.8490880 -4.8143585

O -1.3512630 -2.5444200 -4.1647035

H 0.7580790 -4.6249300 -1.5265795

O 1.1034980 -2.8458510 -3.0947035

C -0.1957830 0.2702330 0.1369575

C -1.1615100 1.2800920 0.2204505

C 0.8918760 0.3170270 1.0262295

C -1.0500240 2.3054190 1.1610805

H -2.0132130 1.2616560 -0.4609445

C 0.9977460 1.3390000 1.9604125

H 1.6645070 -0.4501350 0.9894785

C 0.0295130 2.3532980 2.0452005

H -1.8159050 3.0803590 1.2053795

H 1.8505150 1.3574760 2.6409155

C 0.1682700 3.4544180 3.0615425

H 1.0969410 4.0182070 2.8997645

H -0.6741500 4.1528540 3.0043975

H 0.2091810 3.0443840 4.0795395

E = -1073.19050750, G = -1073.040047

Imaginary frequency = -565.04

**TS 10e**

C 0.3151568 1.1048113 -0.8604033

H 1.2135318 0.9881043 -1.4729703

C -0.4086262 2.2209273 -0.9976983

H -1.3152252 2.4180113 -0.4229633

C -0.0271252 3.3211463 -1.9678673

H 0.9440968 2.9504323 -2.5191873

O 0.3636508 4.5242373 -1.3531243

Cl 0.1578208 3.5580723 -4.6296043

O 1.5004478 3.0579503 -4.0048943

H -0.4319762 5.0489043 -1.1815523

O -0.9335402 3.4759933 -2.9242883

C 0.0305278 -0.0169237 0.0486257

C 0.9186038 -1.0957647 0.0894107

C -1.1011652 -0.0624057 0.8850397

C 0.7102108 -2.1934057 0.9275387

H 1.8015638 -1.0827717 -0.5506823

C -1.3240812 -1.1415147 1.7226527

H -1.8211282 0.7550183 0.8827727

C -0.4188782 -2.2156667 1.7494727

H 1.4263048 -3.0108837 0.9275137

H -2.2005112 -1.1776577 2.3686517

O -0.7256762 -3.2278637 2.6017777

C 0.1705758 -4.3327227 2.6503257

H -0.2520192 -5.0295997 3.3785487

H 0.2398688 -4.8237087 1.6702067

H 1.1675928 -4.0127197 2.9826977
E = -1148.38808242, G = -1148.233163

Imaginary frequency = -586.85

**11a**

C -0.3629805 -0.5335003 -1.0202120

H -0.9409265 -0.1942633 -1.8834860

C 0.0952135 -1.7956983 -1.0380090

H 0.6878925 -2.2186273 -0.2266080

C -0.1910975 -2.6589783 -2.2048030

O 0.3630505 -3.8870593 -2.1888350

H 0.8890785 -4.0262893 -1.3871450

O -0.8729175 -2.3426313 -3.1582280

C -0.1627485 0.4574027 0.0437540

C -0.6771765 1.7486927 -0.1446850

C 0.5210335 0.1654997 1.2361640

C -0.5121925 2.7302587 0.8314580

H -1.2088865 1.9811927 -1.0678860

C 0.6827675 1.1444637 2.2102840

H 0.9277055 -0.8305513 1.4064470

C 0.1677105 2.4296127 2.0110860

H -0.9154435 3.7295337 0.6707990

H 1.2123465 0.9076487 3.1324080

H 0.2975715 3.1932937 2.7774970

E = -498.116161854, G = -498.000122

**11b**

C -0.4295864 -1.1462001 -1.1617092

H -1.0334124 -0.9786551 -2.0561142

C 0.0360826 -2.3835131 -0.9392002

H 0.6477946 -2.6358611 -0.0734522

C -0.2793704 -3.4693271 -1.8995192

O 0.2458676 -4.6774961 -1.6282242

H 0.7803936 -4.6639351 -0.8196372

O -0.9653624 -3.3378581 -2.8905932

C -0.2042604 0.0297699 -0.3086582

C -0.7588514 1.2540089 -0.7114302

C 0.5378826 -0.0297701 0.8835218

C -0.5834534 2.4039879 0.0504588

H -1.3348524 1.3047049 -1.6345732

C 0.7223566 1.1061739 1.6571828

H 0.9775316 -0.9673391 1.2170418

C 0.1560196 2.3033419 1.2228588

H -1.0103284 3.3552699 -0.2563092

H 1.2932426 1.0706159 2.5812998

N 0.3500406 3.5068709 2.0415928

O -0.1417164 4.5520669 1.6548308

O 0.9939816 3.4031439 3.0706328

E = -702.588702942, G = -702.474338

**11c**

C 1.1118576 0.5523204 0.0055073

H 1.4269276 1.5984464 0.0080943

C 2.0704376 -0.3874276 0.0027503

H 1.8476776 -1.4542046 -0.0027837

C 3.4934266 0.0179984 0.0038333

O 4.3984656 -0.9793696 -0.0251257

H 3.9704026 -1.8485686 -0.0487997

O 3.8917336 1.1642434 0.0281553

C -0.3372214 0.3216314 0.0040113

C -1.1892034 1.4349594 -0.0095867

C -0.9042064 -0.9635666 0.0167713

C -2.5738624 1.2837634 -0.0118657

H -0.7620994 2.4377074 -0.0193987

C -2.2828204 -1.1328746 0.0157023

H -0.2701124 -1.8484946 0.0282903

C -3.1009484 -0.0028576 0.0010093

H -3.2307064 2.1512624 -0.0227197

H -2.7202044 -2.1292666 0.0268413

Cl -4.8395434 -0.2157016 -0.0006857

E = -957.712025205, G = -957.608955

**11d**

C -0.3556762 -1.1921373 -1.4116056

H -1.0008992 -0.9746293 -2.2665106

C 0.2310708 -2.4004443 -1.3695966

H 0.8928778 -2.7034153 -0.5583756

C -0.0119042 -3.3708853 -2.4566206

O 0.6353808 -4.5498103 -2.3596476

H 1.1876528 -4.5852193 -1.5642476

O -0.7377452 -3.1857053 -3.4126096

C -0.2219012 -0.1178313 -0.4243276

C -0.8989292 1.0874607 -0.6503786

C 0.5553188 -0.2406253 0.7419564

C -0.8046822 2.1419397 0.2572764

H -1.5055722 1.1984907 -1.5497646

C 0.6440088 0.8116507 1.6415674

H 1.0939848 -1.1645423 0.9486594

C -0.0336932 2.0221887 1.4159344

H -1.3397992 3.0710947 0.0604364

H 1.2497808 0.6998317 2.5417584

C 0.0793498 3.1474687 2.4073434

H 1.1271738 3.4528627 2.5290224

H -0.5002122 4.0195967 2.0855234

H -0.2855852 2.8326597 3.3942064

E = -537.418520447, G = -537.278436

**11e**

C 0.2826846 1.5355665 -1.3841048

H 0.8498636 1.2907155 -2.2858828

C -0.1339244 2.8071535 -1.2431128

H -0.6959294 3.1483605 -0.3734268

C 0.1887066 3.7993825 -2.2869458

O -0.2005434 5.0682595 -2.0458118

H -0.6480874 5.1408245 -1.1897448

O 0.7726736 3.5628155 -3.3261748

C 0.0695246 0.4329565 -0.4494688

C 0.6342276 -0.8145865 -0.7389968

C -0.6764184 0.5660255 0.7389592

C 0.4841316 -1.9061805 0.1158592

H 1.2113566 -0.9368355 -1.6560198

C -0.8384444 -0.5059855 1.5962612

H -1.1358404 1.5198195 0.9953882

C -0.2545304 -1.7491905 1.2928142

H 0.9409466 -2.8570425 -0.1441818

H -1.4143664 -0.4087635 2.5154892

O -0.4620554 -2.7325025 2.2001792

C 0.1468646 -3.9964475 1.9503342

H -0.1033924 -4.6225725 2.8102272

H -0.2550104 -4.4488775 1.0338952

H 1.2375636 -3.8928945 1.8744652
E = -612.616624859, G = -612.471408

***t*-BuOH**

O 0.0510670 -1.4902205 0.7623249

C -0.0035120 -0.1933895 0.1398619

C 1.3814670 0.1707535 -0.3853721

C -0.4381600 0.7638865 1.2395159

C -1.0238050 -0.2224875 -0.9935911

H 2.1134250 0.1643415 0.4324969

H 1.3741260 1.1691475 -0.8417611

H 1.7039250 -0.5510715 -1.1487701

H -0.7215580 -0.9465385 -1.7631351

H -1.1066430 0.7641995 -1.4676181

H -2.0109890 -0.5115935 -0.6107031

H -1.4215820 0.4741085 1.6317459

H -0.5058010 1.7870505 0.8492379

H 0.2856440 0.7520385 2.0646789

H 0.3223960 -2.1302245 0.0910879

E = -233.602764652, G = -233.496822
